# Interleukin-15-Induced CD56^+^ Myeloid Dendritic Cells Combine Potent Tumor Antigen Presentation with Direct Tumoricidal Potential

**DOI:** 10.1371/journal.pone.0051851

**Published:** 2012-12-28

**Authors:** Sébastien Anguille, Eva Lion, Jurjen Tel, I. Jolanda M de Vries, Karen Couderé, Phillip D. Fromm, Viggo F. Van Tendeloo, Evelien L. Smits, Zwi N. Berneman

**Affiliations:** 1 University of Antwerp, Faculty of Medicine and Health Sciences, Vaccine and Infectious Disease Institute (VAXINFECTIO), Laboratory of Experimental Hematology, Antwerp, Belgium; 2 Antwerp University Hospital, Center for Cell Therapy and Regenerative Medicine, Antwerp, Belgium; 3 Radboud University Nijmegen Medical Centre and Nijmegen Centre for Molecular Life Sciences, Department of Tumor Immunology, Nijmegen, The Netherlands; 4 ANZAC Research Institute, Dendritic Cell Biology and Therapeutics Group, Sydney, Australia; Centre de Recherche Public de la Santé (CRP-Santé), Luxembourg

## Abstract

Dendritic cells (DCs) are the quintessential antigen-presenting cells of the human immune system and play a prime role in coordinating innate and adaptive immune responses, explaining the strong and still growing interest in their application for cancer immunotherapy. Much current research in the field of DC-based immunotherapy focuses on optimizing the culture conditions for *in vitro* DC generation in order to assure that DCs with the best possible immunogenic qualities are being used for immunotherapy. In this context, monocyte-derived DCs that are alternatively induced by interleukin-15 (IL-15 DCs) have attracted recent attention due to their superior immunostimulatory characteristics. In this study, we show that IL-15 DCs, in addition to potent tumor antigen-presenting function, possess tumoricidal potential and thus qualify for the designation of killer DCs. Notwithstanding marked expression of the natural killer (NK) cell marker CD56 on a subset of IL-15 DCs, we found no evidence of a further phenotypic overlap between IL-15 DCs and NK cells. Allostimulation and antigen presentation assays confirmed that IL-15 DCs should be regarded as *bona fide* myeloid DCs not only from the phenotypic but also from the functional point of view. Concerning their cytotoxic activity, we demonstrate that IL-15 DCs are able to induce apoptotic cell death of the human K562 tumor cell line, while sparing tumor antigen-specific T cells. The cytotoxicity of IL-15 DCs is predominantly mediated by granzyme B and, to a small extent, by tumor necrosis factor-α (TNF-α)-related apoptosis-inducing ligand (TRAIL) but is independent of perforin, Fas ligand and TNF-α. In conclusion, our data provide evidence of a previously unappreciated role for IL-15 in the differentiation of human monocytes towards killer DCs. The observation that IL-15 DCs have killer DC capacity lends further support to their implementation in DC-based immunotherapy protocols.

## Introduction

Over the past years, the phenotypic and functional boundaries distinguishing the main cell subsets of the human immune system have become increasingly blurred. While it has already been well established that T cells may share some phenotypic and functional features with natural killer (NK) cells [1], more recent evidence also points to the existence of such overlap between NK cells and dendritic cells (DCs) [2]. NK cells have been shown capable of antigen presentation, a classical function of DCs [3]. In mice, specialized NK cell subsets, collectively designated as ‘natural killer dendritic cells’ (NKDCs), have been identified that display a hybrid NK cell/DC phenotype and combine functional properties of NK cells (cytotoxicity) and DCs (antigen presentation) [4]–[9]. Conversely, evidence from both rodent and human studies is emerging that DCs may exhibit NK-like activity and play a direct role in innate immunity as killer cells; in the literature, these cells are designated as ‘killer DCs’ [10]–[13]. Such killer DCs that can combine both tumor antigen presentation function with direct tumoricidal activity are garnering increasing attention as potential new, multifunctional tools for cancer immunotherapy [10]–[12], [14].

Hitherto, monocyte-derived DCs represent the DC type most widely used in human immunotherapy trial protocols [15], [16]. They are classically obtained through *in vitro* differentiation of peripheral blood monocytes in the presence of granulocyte macrophage colony-stimulating factor (GM-CSF) and interleukin (IL)-4 [17], followed by induction of DC maturation using a pro-inflammatory cytokine cocktail composed of tumor necrosis factor (TNF)-α, IL-1β, IL-6 and prostaglandin E_2_ (PGE_2_) [18]. Over the years, it has become apparent that these “gold-standard” DCs, commonly referred to as ‘IL-4 DCs’, are suboptimal in terms of antigen presentation function and T cell stimulatory capacity [17]. This explains the impetus behind the many efforts that are currently being made to optimize the culture conditions for *ex vivo* monocyte-derived DC generation [19], [20].

Within this context, we and others have shown that the immunostimulatory properties of monocyte-derived DCs can be significantly enhanced by replacing IL-4 with IL-15 for DC differentiation and by using Toll-like receptor (TLR) stimuli to trigger DC maturation [17], [21]–[23]. In addition, we have found that these so-called ‘IL-15 DCs’ display a rather unconventional DC phenotype, with a subset of these cells being positive for the cell surface marker CD56 [17]. Since CD56 is the archetypal phenotypic marker of NK cells, we here aimed to investigate whether IL-15 DCs also bear functional resemblance with NK cells in terms of cytotoxic activity. In this study, IL-15 DCs are shown to possess potent tumor antigen presentation function in combination with lytic potential against the classical NK cell target cell line K562, thus confirming the hypothesis that IL-15 DCs qualify for the designation of killer DCs.

## Methods

### Ethics statement

This study was approved by the Ethics Committee of the University of Antwerp (Antwerp, Belgium) under the reference number 11/47/366. All experiments were performed using blood samples from anonymous volunteer donors, provided through the Antwerp Blood Transfusion Center of the Red Cross (Edegem, Belgium).

### Human cell lines

The human myeloid leukemia cell lines K562 and U937 were obtained from American Type Culture Collection (Rockville, MD, USA) and maintained in Iscove's modified Dulbecco's medium (IMDM; Invitrogen, Merelbeke, Belgium) supplemented with 10% fetal bovine serum (FBS; Invitrogen). The human cytotoxic T lymphocyte (CTL) clone specific for the HLA-A*0201-restricted epitope 126–134 of the Wilms' tumor 1 protein (WT1) [24] was kindly provided by Dr C. Bonini (San Raffaele Scientific Institute, Milan, Italy) and maintained in IMDM/10% FBS and 60 IU/mL IL-2 (Immunotools, Friesoythe, Germany).

### DC culture

Mature IL-15 DCs were generated according to our previously described protocol [17]. Briefly, peripheral blood mononuclear cells (PBMCs) were prepared from buffy coats by standard Ficoll density gradient centrifugation (Ficoll-Paque™ PLUS; GE Healthcare, Diegem, Belgium). CD14^+^ monocytes were purified using a positive immunomagnetic cell selection kit (Miltenyi, Amsterdam, The Netherlands) and seeded into 6-well culture plates (Corning Life Sciences, Schiphol-Rijk, The Netherlands) at a density of 1–1.2×10^6^/mL in DC differentiation medium containing Roswell Park Memorial Institute medium (RPMI-1640; Invitrogen), 2.5% heat-inactivated human AB serum (Invitrogen), 800 IU/mL GM-CSF (Invitrogen) and 200 ng/mL IL-15 (Immunotools). After 24–36 hr of *in vitro* culture, CD56^+^ and CD56^−^ IL-15 DC populations were separated using anti-CD56 magnetic microbeads according to the manufacturer''s instructions (Miltenyi). Both fractions were resuspended in DC differentiation medium and cultured for an additional 16–20 hr in the presence of a DC maturation cocktail containing 3 μg/mL of the TLR7/8 ligand R-848 (Enzo Life Sciences, Antwerp, Belgium), 2.5 ng/mL TNF-α (Gentaur, Brussels, Belgium), 5000 IU/mL interferon-γ (IFN-γ; Immunotools) and 1 μg/mL PGE_2_ (Pfizer, Puurs, Belgium). In some experiments, IL-15 DC function was compared to that of conventional 7-day GM-CSF/IL-4 monocyte-derived DCs (IL-4 DCs), which were generated as described previously [17].

### Flow cytometric immunophenotyping

Purity of IL-15 DC cultures was checked on a routine basis by multiparameter flow cytometry using a combination of FITC-, PE-, PB-/V450- and APC-conjugated monoclonal antibodies (mAbs) specific for CD3, CD7, CD11c, CD19 and CD56. All mAbs were from Becton Dickinson (BD; Erembodegem, Belgium) unless specified otherwise. Cell surface staining of mature IL-15 DCs was performed using FITC-, PE-, PB-/V450-, or APC-conjugated mAbs against various NK cell markers (CD7, CD16, CD56, CD69, NKG2D [Miltenyi], NKp46), NKDC-associated surface antigens (CD11c, B220 [eBioscience, Halle-Zoersel, Belgium], NKR-P1A/CD161 [Miltenyi]) and monocyte/DC markers (BDCA-1/CD1c [Miltenyi], CD14, CD40, CD80, CD83 [Invitrogen], CD86, CD209/DC-SIGN, CCR7 [R&D Systems, Minneapolis, MN, USA], HLA-DR). For detection of membrane-bound cytolytic effector molecules, DCs were stained with PE-labeled mAbs against TNF-α, Fas ligand/CD178 (eBioscience) and TNF-α-related apoptosis-inducing ligand (TRAIL). Dead cells were excluded by 7-amino actinomycin D (7-AAD; 5 µL/sample; BD) staining 10 min prior to acquisition on a FACSAria II (BD) flow cytometer. In each experiment, isotype-matched control mAbs were included to determine non-specific background staining. Results were expressed as delta mean fluorescence intensity (ΔMFI) (calculated by subtracting MFI values of isotype controls from sample MFI values) and as percentages of marker-positive cells (determined by Overton subtraction of isotype control histograms from sample histograms).

### CD56 expression kinetics

In a separate experiment designed to study the kinetics of CD56 expression during IL-15 DC differentiation, ultra-purified FACS-sorted monocytes were used as precursor cells for IL-15 DC differentiation. For this specific experiment, CD14 magnetic bead-selected monocytes were stained with CD14-PerCP-Cy5.5/CD16-V500 (both from BD) and then flow-sorted using an Influx cytometer (BD). Sorting gates were set conservatively on CD14^++^CD16^−^ cells to ensure high-purity isolation (>99.9%) of the “classical” monocyte subset. Sorted monocytes were then subjected to IL-15 DC differentiation as described above. Flow cytometric analysis of CD56 expression on IL-15 DCs was performed at different time points following start of culture (4 hr, 16 hr, 24 hr, 40 hr, 48 hr, 64 hr, 72 hr, 96 hr and 7 days) by double surface staining with CD11c-APC (BD) and CD56-PB (BioLegend, San Diego, CA, USA). At each time point, samples were stained in parallel with CD11c-APC (BD) and a PB-labeled isotype-matched control mAb (mouse IgG2a, κ; BioLegend) to determine non-specific background labeling. Samples were acquired on an Influx cytometer (BD).

### Combined cell surface and intracellular staining

Brefeldin A (1 µL/10^6^ cells; Invitrogen) was added to the DC cultures 180 min prior to harvesting the cells. After harvest, cells were surface-stained with CD11c-V450 and CD56-APC (both from BD). The LIVE/DEAD® fixable violet stain with 405 nm excitation (1 µL/10^6^ cells; Invitrogen) was concomitantly added to allow discrimination between viable and non-viable cells. After 30 min incubation at room temperature, cells were treated sequentially with FACS lysing solution and FACS permeabilizing solution 2 (BD). Intracellular expression of cytolytic effector molecules was assessed after 4–6 hr incubation at 4°C with one of the following mAbs or their corresponding isotype controls: perforin-FITC, granzyme B-PE or TRAIL-PE (all from BD). Samples were acquired using a FACSAria II cytometer (BD). Results were expressed as ΔMFI values, as specified above.

### Granzyme B secretion

CD56^+^ and CD56^−^ IL-15 DCs were harvested 16–20 hours following addition of the maturation cocktail, washed, and resuspended in IMDM/10% FBS at a density of 2×10^6^ viable cells/mL. Cell-free culture supernatants were collected after overnight incubation and cryopreserved at −20°C for later analysis. Thawed supernatants were diluted 5-fold to allow quantification of granzyme B using a commercially available ELISA kit according to the manufacturer's instructions (Diaclone, Besançon, France).

### Allogeneic mixed lymphocyte reaction (allo-MLR)

To prepare the responder cells for the MLR, frozen CD14^+^ monocyte-depleted PBMCs of an allogeneic blood donor were thawed and further depleted of NK cells and NKT cells using anti-CD56 magnetic microbeads (Miltenyi). The resultant lymphocyte fraction was then labeled with 5,6-carboxyfluorescein diacetate succinimyl ester (CFSE; 5 µM; Invitrogen), and co-cultured with mature CD56^+^ and CD56^−^ IL-15 DCs in a 96-well round-bottom plate (Corning) at a stimulator:responder ratio of 1∶10. Unstimulated CFSE-labeled cells served as negative control, while CFSE-labeled responders cells stimulated by either IL-4 DCs or a combination of phytohemagglutinin (PHA; 1 µg/mL; Sigma-Aldrich, Bornem, Belgium) and IL-2 (20 IU/mL; Immunotools) served as positive controls. After 5 days, cells were stained with CD11c-V450, CD3-APC and CD4-APC-H7 (all from BD) and analyzed using a FACSAria II cytometer (BD). T cell proliferation was assessed by quantifying the percent of CFSE-diluted (CFSE^low^) cells within the CD11c^−^CD3^+^CD4^+^ gate after background subtraction.

### Antigen presentation assay

To evaluate their capacity for tumor antigen processing and presentation, IL-15 DCs were loaded by electroporation with *in vitro* transcribed *WT1*-encoding RNA. *WT1* RNA was produced by eTheRNA (Prof K. Thielemans, Free University of Brussels, Brussels, Belgium) starting from a T7 promotor-driven plasmid containing the codon-optimized (GeneArt, Regensburg, Germany) human *WT1* gene, lacking its 5′-,3′-untranslated regions as well as the aa292–348 nuclear localization sequence and flanked at its 5′- and 3′-sides, respectively, by the signal sequence and the HLA class II-targeting sequence of DC-lysosome-associated membrane protein [20]. IL-15 DCs from HLA-A*0201^+^ individuals were prepared and electroporated as previously described [17], with minor modifications. Briefly, 5×10^6^ cells were transferred to a 4-mm electroporation cuvette (Bio-Rad, Hercules, CA, USA) and electrotransfected with 20 µg RNA by an exponential decay pulse of 300 V/300 µF using the Gene Pulser Xcell device (Bio-Rad). Four hours post-electroporation, cells were transferred to 96-well round-bottom plates (Corning) and co-cultured in triplicate with the WT1_126–134_-specific CTL clone at a DC-to-T cell ratio of 1∶1. The next day, supernatants were collected and analyzed for IFN-γ production by ELISA as per the manufacturer's instructions (Peprotech, Rocky Hill, NJ, USA). In parallel experiments, *WT1* RNA-electroporated IL-15 DCs were co-cultured with the CTL clone at a DC-to-T cell ratio of 40∶1 in triplicate wells of anti-IFN-γ-antibody-coated 96-well PVDF-bottom ELISpot plates (Millipore, Bedford, MA, USA). After overnight co-culture at 37°C, plates were developed using a commercially available IFN-γ ELISpot kit (Diaclone). IFN-γ spot-forming cells (SFCs) were counted with a computerized ELISpot plate reader (Autoimmun Diagnostika, Strassberg, Germany). In each assay, the following controls were included to determine the levels of non-antigen-specific IFN-γ production: T cells cultured alone, DCs cultured alone and T cells cultured with non-antigen-loaded DCs.

### Cytotoxicity assays

A flow cytometry-based lysis assay [25] was used to determine the cytotoxic activity of IL-15 DCs against the following potential targets: the K562 human myeloid leukemia cell line (major histocompatibility complex [MHC] class I-negative), the U937 human myeloid leukemia cell line (MHC class I-positive) and the WT1_126–134_-specific CTL clone. Briefly, target cells were labeled with the PKH67 green fluorescent cell linker (Sigma-Aldrich). DCs were harvested following maturation, rigorously washed, and co-cultured overnight with PKH67-labeled target cells in fresh IMDM/10% FBS medium at different effector-to-target (E:T) ratios (50:1, 25:1, 12:1, 6∶1 and 1∶1). Prior to flow cytometric measurement, the cell surface was stained with CD11c-V450 (BD) to allow further discrimination between effector cells (PKH67^−^CD11c^+^) and targets cells (PKH67^+^CD11c^−^). The cells were then washed and resuspended in Annexin-V-binding buffer (BD) containing Annexin-V-APC (1 µL/100 µL buffer; BD). After 15 min incubation at room temperature, samples were stained with propidium iodide (PI; Sigma-Aldrich) and immediately acquired on a Partec CyFlow ML (Partec, Münster, Germany) or a FACSAria II (BD) multiparameter flow cytometer. PKH67-labeled target cells cultured without DCs served as controls to determine spontaneous cell death. Percentages of viable target cells (i.e. percentages of PI^−^/Annexin-V^−^ cells within the PKH67^+^/CD11c^−^ target cell gate) were used to quantify the cytotoxic responses according to the following formula: % of specific lysis  = 100– [(% viable target cells in the presence of DCs/% viable target cells without DCs) ×100].

### Cytotoxicity blocking studies

Cytotoxicity blocking experiments were performed as described above, except that effector cells (CD56^+^ IL-15 DCs) were pre-incubated at 37°C with either neutralizing TRAIL mAbs (40 µg/10^6^ cells; R&D Systems) or concanamycin A (200 nM/10^6^ DCs; Tocris Bioscience, Bristol, UK) before addition of PKH67-labeled K562 target cells at a 50:1 E:T ratio. Control experiments were run in parallel using TRAIL isotype control mAbs (mouse IgG1; R&D Systems) or concanamycin A control medium, respectively.

### Data mining and statistical analysis

Flow cytometry data were analyzed using FlowJo software (v9.3; Treestar, Ashland, OR, USA). GraphPad Prism software (v5.0; San Diego, CA, USA) was used for statistical analysis and graphing. Statistical comparisons were performed using Wilcoxon's matched-pairs signed rank test or paired Student's *t*-test, where appropriate. *P*-values <0.05 were considered statistically significant. All data were expressed as means ± standard error of the mean (SEM).

## Results

### IL-15 DCs are CD56^+/−^ myeloid DCs that are phenotypically unrelated to NK cells

**Figure 1:**
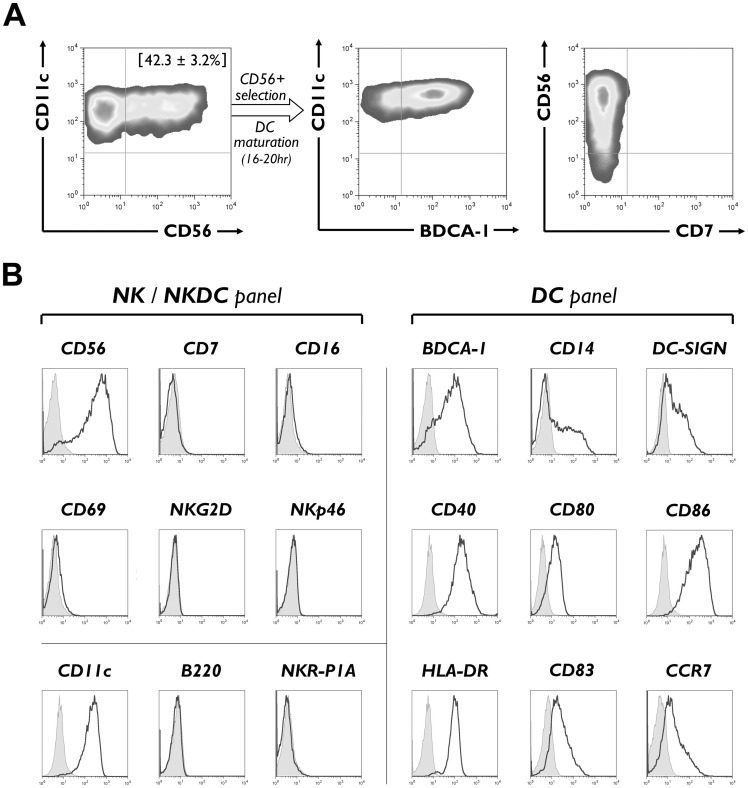
Phenotypic characteristics of CD56^+^ IL-15 DCs. (A) CD14^+^ monocytes were cultured for 24–36 hr in the presence of GM-CSF and IL-15 (IL-15 DCs) and analyzed by flow cytometry for expression of CD11c/CD56 (left). The percentage between parentheses indicates the mean (± SEM) percentage of CD56^+^ cells among the total IL-15 DC population (*n* = 17). These CD56^+^ cells were then immunomagnetically separated, cultured for another 16–20 hr in the presence of DC maturation cocktail and analyzed for co-expression of CD11c/BDCA-1 (middle) and CD56/CD7 (right). Quadrant gates were set using corresponding isotype controls. (B) Matured CD56^+^ IL-15 DCs were further analyzed by flow cytometry for expression of the indicated NK cell-associated (CD56, CD7, CD16, CD69, NKG2D, NKp46), NKDC-associated (CD11c, B220, NKR-P1A) and DC-related surface antigens. Histogram overlays show expression of the indicated markers (solid line histograms) compared to their respective isotype controls (filled grey histograms). All plots are representative of at least 4 independent experiments.

Exposure of human peripheral blood CD14^+^ monocytes to GM-CSF and IL-15 resulted in their rapid differentiation into CD11c^+^ DCs with subset expression of CD56 (Figure 1A, left panel). Acquisition of CD56 was also demonstrable on IL-15 DCs differentiated from ultra-purified, FACS-sorted CD14^+^ monocytes, with CD56 being detectable already within the first 24 hr of culture. Maximal CD56 surface expression was observed between 24–48 hr after start of IL-15 DC culture, after which expression gradually declined toward the non-specific background level at day 7 (Figure S1).

Further phenotypic analysis of the CD56^+^ IL-15 DC fraction revealed that this subset displayed the characteristic forward scatter (FSC)/sideward scatter (SSC) profile of DCs (data not shown) and that it co-expressed CD11c and BDCA-1, indicating a myeloid DC phenotype (Figure 1A, middle panel). In addition, CD56^+^ IL-15 DCs were found to lack co-expression of CD7, which confirmed that these cells are unrelated to NK cells (Figure 1A, right panel) [26]. Natural cytotoxicity receptors (NCRs), such as the NK cell-specific marker NKp46 [27], and other typical NK cell-associated markers, such as CD16, CD69 or NKG2D, were not expressed on the cell surface of CD56^+^ IL-15 DCs (Figure 1B, ‘NK panel’).

The co-expression of CD11c with the prototypical NK cell marker CD56 led us to further investigate whether the phenotype of CD56^+^ IL-15 DCs might correspond to that of the so-called NKDCs in mice [5]–[7]. Notwithstanding expression of CD11c, we were unable to classify CD56^+^ IL-15 DCs as their human counterpart due to the lack of NKDC-associated surface hallmarks such as NKR-P1A/CD161, which is the human homologue of murine NK1.1 [28], and B220 (Figure 1B, ‘NKDC panel’).

After 2–3 days of culture, including a maturation step during the final 16–20 hr, both CD56^+^ and CD56^−^ IL-15 DC populations developed a mature DC phenotype characterized by a marked down-regulation of CD14, up-regulation of DC-SIGN, high-level expression of co-stimulatory molecules (CD40, CD80 and CD86) and of HLA-DR as well as of the maturation markers CD83 and CCR7 (Table 1 and Figure 1B, ‘DC panel’). Compared to their CD56^−^ counterparts, CD56^+^ IL-15 DCs showed more prominent down-regulation of CD14 along with significantly higher expression levels of CD40, CD80, CD86, CD83 and CCR7, indicative of a more differentiated and/or activated DC phenotype (Table 1).

**Table 1:** Phenotypic difference between matured CD56^+^ and CD56^−^ IL-15 DCs.

	ΔMFI		% positive cells	
	CD56^−^ IL-15 DCs	CD56^+^ IL-15 DCs	CD56^−^ IL-15 DCs	CD56^+^ IL-15 DCs
**CD11c**	262.6±40.1	364.0±51.5*	98.5±0.6	99.0±0.6*
**BDCA-1/CD1c** (n = 4)	19.1±5.3	39.7±9.3	60.0±4.4	74.5±4.0
**CD14** (n = 6)	16.3±4.2	10.9±3.5*	51.5±5.8	40.5±5.4*
**DC-SIGN/CD209**	4.7±1.1	6.0±1.6	40.9±4.4	44.0±5.4
**CD40**	169.7±21.8	227.6±23.6*	97.9±0.9	98.9±0.5*
**CD80**	6.8±1.3	9.3±1.8*	59.7±4.8	65.0±4.8
**CD86**	168.4±21.3	223.5±28.9*	97.1±0.6	98.1±0.5*
**HLA-DR**	106.3±12.9	107.0±13.9	96.5±0.8	96.0±1.1
**CCR7**	7.2±3.1	11.1±3.7*	35.8±8.7	46.4±8.8*
**CD83**	13.8±3.6	19.1±5.4*	62.3±6.4	68.1±5.7*

Pairwise comparison of CD56^−^ and CD56^+^ IL-15 DC surface phenotypes. Results are presented as mean (± SEM) ΔMFI values and as mean (± SEM) percentage of cells staining positive for the indicated cell surface marker. Data are from 7 independent experiments, except if specified otherwise. Asterisks indicate a statistically significant difference in cell surface marker expression between CD56^+^ and CD56^−^ IL-15 DC populations (*P*<0.05).

### IL-15 DCs possess allo-stimulatory capacity

**Figure 2:**
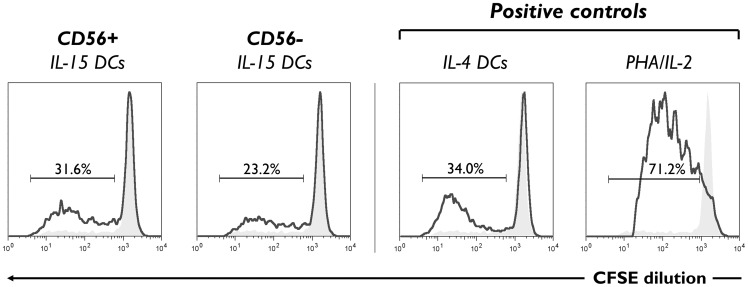
Allostimulatory capacity of CD56^+^ and CD56^−^ IL-15 DCs in a MLR. CD56^+^ and CD56^−^ IL-15 DCs were co-cultured with allogeneic, CFSE-labeled lymphocytes at a 1∶10 ratio for 5 days. CFSE-labeled cells stimulated with IL-4 DCs (1∶10 stimulator/responder ratio) or PHA/IL-2 were used as positive controls. Histograms show the degree of CFSE dilution, indicative of T cell proliferation, among gated CD3^+^CD4^+^ T cells in the absence (filled grey histograms) and presence (solid line histograms) of the indicated stimulators. Numbers above the bracketed lines indicate the background-subtracted percentages of proliferated (i.e. CFSE^low^) T cells within the CD3^+^CD4^+^ gate. Data shown are representative of three donors.

Next, CD56^+^ and CD56^−^ IL-15 DC subpopulations were subjected to an allo-MLR in order to examine their ability to stimulate allogeneic T cell proliferation, one of the defining functional characteristics of DCs [29]. While NK cells were unable to stimulate allogeneic T cell proliferation (data not shown), IL-15 DCs were found to possess potent allostimulatory capacity, as determined by CFSE dilution analysis (Figure 2). There was no statistically significant difference between the percent of CFSE^low^ CD4^+^ T cells in CD56^+^ as compared to CD56^−^ IL-15 DC-stimulated cultures (background-subtracted % CFSE^low^ CD4^+^ T cells following stimulation with CD56^+^ *vs.* CD56^−^ DCs: 24.1±3.9% vs. 19.7±2.6%; *P>*0.05).

### IL-15 DCs are functional DCs with potent tumor antigen-presenting capacity

**Figure 3:**
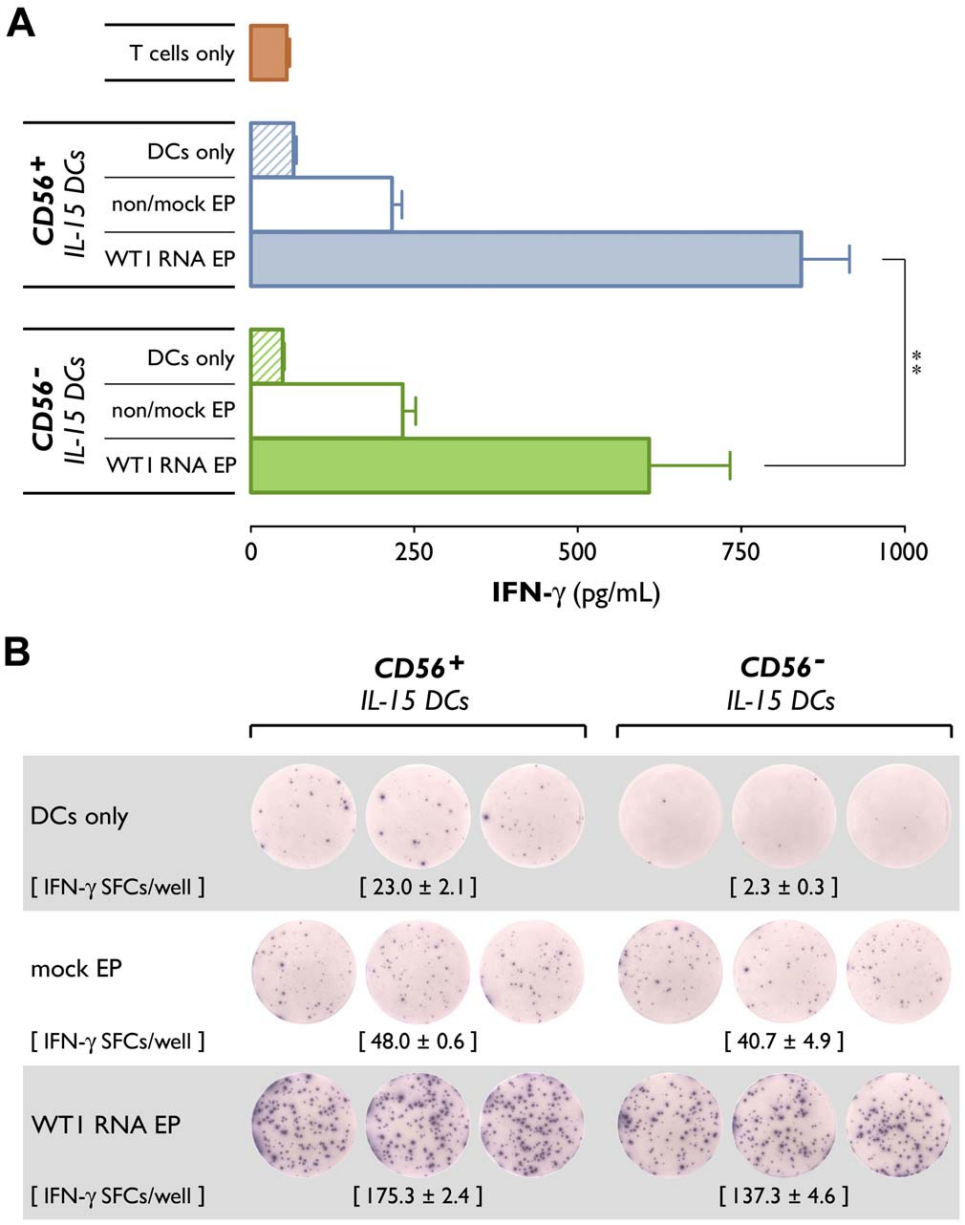
Differential ability of CD56^+^ and CD56^−^ IL-15 DCs to stimulate a WT1-specific CTL clone. Mature CD56^+^ and CD56^−^ IL-15 DCs were electroporated (EP) with *WT1* RNA and co-cultured with an HLA-A*0201-restricted WT1_126–134_-specific CTL clone. Negative controls included: T cells cultured without DCs (T cells only), *WT1* RNA-electroporated DCs cultured without T cells (DCs only), and T cells cultured with non-antigen-loaded DCs (non/mock EP). (A) After overnight incubation, IFN-γ concentrations (pg/mL) in the culture supernatants were measured by ELISA. Bars represent mean (± SEM) IFN-γ concentrations of triplicate wells of three independent experiments (**, *P* = 0.003). (B) Antigenic responses were quantified in parallel by IFN-γ ELISpot. Illustrative single-well images from an IFN-γ ELISpot plate of one representative experiment are shown. Values in parentheses indicate the mean (± SEM) number of IFN-γ spot-forming cells (SFCs) of triplicate ELISpot wells.

To determine their capacity for tumor antigen presentation, IL-15 DCs were loaded by RNA electroporation with the WT1 tumor antigen [30] and examined for their ability to trigger IFN-γ production by a WT1_126–134_-specific CTL clone. *WT1* RNA-electroporated IL-15 DCs elicited a marked increase in IFN-γ secretion, as determined by ELISA, indicating that RNA translation and processing for MHC class I presentation had occurred in these cells. Production of IFN-γ in response to non-antigen-loaded IL-15 DCs was approximately 3.3-fold lower as compared to *WT1* RNA-electroporated IL-15 DCs, confirming the antigen specificity of the responses observed (Figure 3A; *WT1* RNA EP *vs.* non/mock EP: *P*<0.001 and *P* = 0.01 for CD56^+^ and CD56^−^ IL-15 DCs, respectively). *WT1* RNA-electroporated CD56^+^ IL-15 DCs were significantly more efficient at antigen presentation than their CD56^−^ counterparts, as was demonstrated by their superior capacity to stimulate the CTL clone to secrete IFN-γ (Figure 3A, *WT1* RNA EP CD56^+^ *vs.* CD56^−^ IL-15 DCs: *P* = 0.003).

Results of the IFN-γ ELISpot assay were similar to those obtained by ELISA. As shown in Figure 3B, both CD56^+^ and CD56^−^ *WT1* RNA-electroporated IL-15 DCs were able to induce robust IFN-γ ELISpot responses. Numbers of IFN-γ SFCs/well in non-antigen-stimulated cultures (i.e. CTL clone cultured with non-antigen-loaded DCs) did not differ significantly between CD56^+^ and CD56^−^ IL-15 DCs and were found to be consistently lower than those in corresponding cultures stimulated with *WT1* RNA-electroporated IL-15 DCs (Figure 3B; *WT1* RNA EP *vs.* non/mock EP: *P*<0.001 for both CD56^+^ and CD56^−^ IL-15 DCs). Although *WT1* RNA-electroporated CD56^−^ IL-15 DCs induced a potent IFN-γ ELISpot response (SFCs/well: 141.4±3.3), significantly higher numbers of IFN-γ SFCs/well (184.4±8.0) were observed when *WT1* RNA-electroporated CD56^+^ IL-15 DCs were used as effectors (*P*<0.001), confirming the superiority of CD56^+^ over CD56^−^ IL-15 DCs in terms of antigen-presenting capacity (Figure 3B).

### IL-15 DCs are killer DCs capable of tumor lysis while sparing antigen-specific T cells

**Figure 4:**
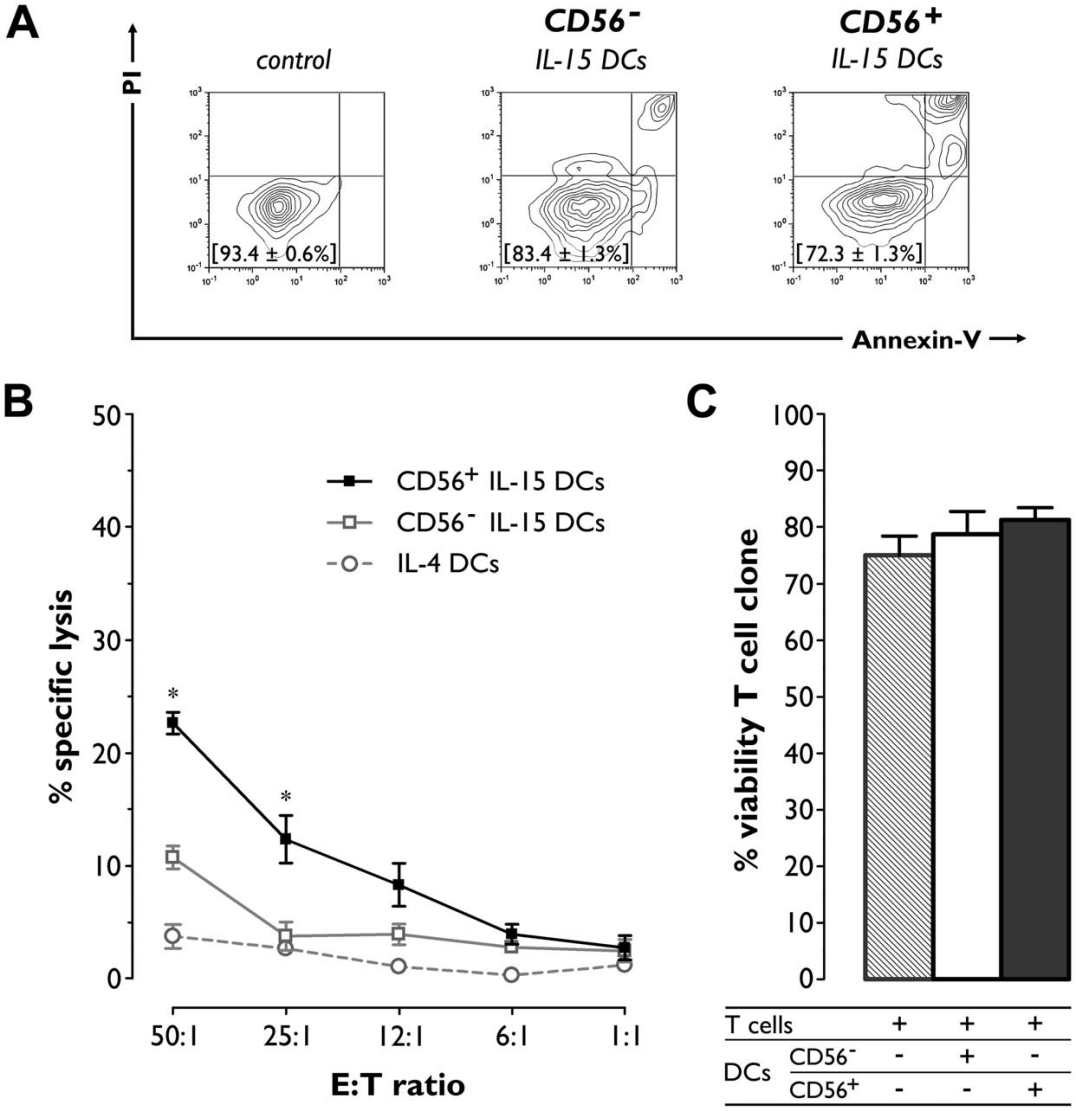
Lysis of K562 cells but not of a WT1-specific CTL clone by IL-15 DCs. PKH67-labeled target cells were mixed at varying E:T ratios with mature CD56^+^ and CD56^−^ IL-15 DCs or, where indicated, with conventionally generated IL-4 DCs and then subjected to PI/Annexin-V staining after overnight incubation. Target cell viability was defined as the percentage of PI^−^/Annexin-V^−^ cells within the PKH67^+^CD11c^−^ gate. (A) Viability profiles of gated K562 tumor cells cultured alone (control) or with either CD56^−^ or CD56^+^ IL-15 DCs at an E:T ratio of 50:1. One representative experiment out of five is shown. Percentages of viable K562 cells are displayed in the lower left quadrants and expressed as mean (± SEM) of 5 independent experiments. (B) Graph depicting the specific lysis of K562 tumor cells by CD56^+^ IL-15 DCs (solid black line, ▪; *n* = 5), CD56^−^ IL-15 DCs (solid grey line, □; *n* = 5) and IL-4 DCs (dashed grey line, ○; *n* = 3) at the indicated E:T ratios. Results are expressed as mean (± SEM) percentages of specific lysis. Asterisks refer to a statistically significant difference in cytotoxic activity at the indicated E:T ratio between CD56^+^ and CD56^−^ IL-15 DCs. (C) Bar graphs showing the viability of a WT1_126–134_-specific CTL clone after overnight culture in the absence or presence of either CD56^−^ (□) or CD56^+^ (▪) IL-15 DCs at an E:T ratio of 50:1. Data are presented as mean (± SEM) percentages of viable T cells from three experiments.

The observation that a subset of IL-15 DCs expressed the classical NK cell marker CD56 prompted us to examine whether these DCs have cytolytic effector function. Therefore, both CD56^+^ and CD56^−^ IL-15 DC fractions were co-cultured with the K562 tumor cell line and examined for lytic activity using a PI/Annexin-V-based flow cytometric lysis assay. After 16–18 hr of co-culture, CD56^+^ IL-15 DCs were found to have reduced K562 cell viability by 22.7±1.0% at an E:T ratio of 50:1 (Figure 4). This decrease in K562 cell viability was due to an increase in apoptotic cell death, as indicated by the higher frequency of PI^+^Annexin-V^+^ K562 target cells after co-culture with CD56^+^ IL-15 DCs (Figure 4A). As shown in Figure 4B, the cytotoxic action of CD56^+^ IL-15 DCs was titratable and most apparent at high E:T ratios, where levels of cytotoxicity between CD56^+^ and CD56^−^ IL-15 DCs were found to differ significantly (Figure 4B; *P*<0.001 and *P* = 0.006 for E:T ratios of 50:1 and 25:1, respectively). At these E:T ratios, lytic activity of CD56^−^ IL-15 DCs was markedly lower as compared to that of CD56^+^ DCs, but still higher than that of conventional IL-4 DCs which failed to induce any significant cytotoxicity (Figure 4B).

We next sought to determine whether IL-15 DCs display any non-specific cytotoxic activity against tumor antigen-specific T cells, which would be unwanted in the context of immunotherapy. To this end, mature CD56^+^ and CD56^−^ IL-15 DCs derived from HLA-A*0201^+^ donors were added to the WT1_126–134_-specific CTL clone at a 50:1 E:T ratio. Residual cell viability was determined after overnight incubation by PI/Annexin-V staining. Figure 4C shows that there was no significant difference in T cell clone viability regardless of the presence or absence of IL-15 DCs, confirming that IL-15 DCs lack lytic activity against WT1-specific T cells (Figure 4C; T cells *vs.* T cells + DCs: *P>*0.05 for both CD56^+^ and CD56^−^ IL-15 DCs).

### Lysis by IL-15 DCs is not due to contamination by cytotoxic lymphocytes

IL-15 DC cultures were routinely checked for purity by multiparameter flow cytometry in order to exclude the possibility that cytotoxicity results were confounded by culture contamination with cytotoxic lymphocytes. As mentioned above, IL-15 DCs could be clearly distinguished from residual lymphocyte subsets on the basis of their distinct FSC/SSC profile, their intense uniform expression of CD11c and lack of CD7 expression. Both CD56^+^ and CD56^−^ IL-15 DC preparations were found to contain minute numbers of contaminating lymphocytes. The percentage of CD7^+^ cells within the contaminating lymphocyte cluster, which includes NK cells and other cytotoxic lymphocytes [26], was 0.9±0.2% for CD56^+^ IL-15 DC cultures and 1.1±0.3% for CD56^−^ IL-15 DC cultures (n = 12).

To further address the possibility that the observed lytic activity against K562 cells might have resulted from this low-level contamination with NK cells, we additionally performed a cytotoxicity assay against the U937 cell line, another known NK cell-sensitive target cell line [25], [31]. As shown in Figure S2, both CD56^+^ and CD56^−^ IL-15 DC preparations failed to affect the viability of U937 cells, even at the high 50:1 E:T ratio used, indicating that the presence of these few NK cell contaminants was not a major concern in our experimental design.

### IL-15 DCs contain TRAIL, express and secrete granzyme B but lack perforin

**Figure 5:**
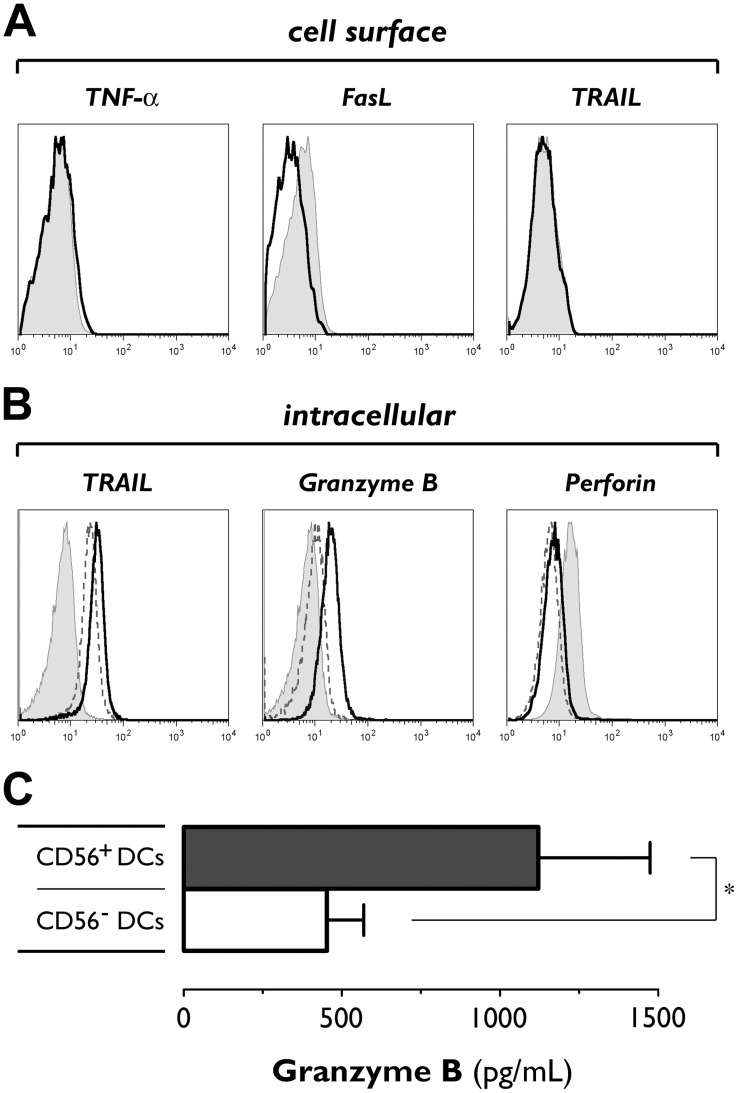
Expression of lytic molecules by IL-15 DCs. (A) Matured CD56^+^ IL-15 DCs were analyzed by flow cytometry for cell surface expression of TNF-α, FasL and TRAIL (solid line histograms). Filled grey histograms represent isotype controls. Data are from one experiment representative of three. (B) Both CD56^+^ (solid line) and CD56^−^ (dashed line) IL-15 DCs were stained for intracellular expression of TRAIL, granzyme B and perforin. Filled grey histograms represent isotype controls. One representative experiment out of 6 (for TRAIL) and 9 (for granzyme B and perforin) is shown. (C) Supernatants from overnight washout cultures of CD56^+^ (▪) and CD56^−^ (□) IL-15 DCs were analyzed for granzyme B release by ELISA. Bars represent mean (± SEM) granzyme B concentations (pg/mL) from 7 experiments.*, *P*<0.05.

The logical next step was to determine the mechanisms that underlie the killing activity of IL-15 DCs. In view of the above observation that the cytotoxic action of these cells relies primarily on the induction of apoptosis, we focused on the most common apoptotic cell death pathways. As shown in Figure 5A, we found no evidence for cell surface expression of the apoptotic death receptor ligands TNF-α, FasL or TRAIL. We next performed intracellular staining experiments to examine the possible involvement of intracellular lytic molecules. In these experiments, DCs were identified on the basis of their characteristic FSC/SSC profile and CD11c positivity. Interestingly, both CD56^+^ and CD56^−^ IL-15 DCs contained intracellular TRAIL protein, which was found to be expressed at a significantly higher level in the CD56^+^ IL-15 DC subset (Figure 5B; ΔMFI for intracellular TRAIL in CD56*^+^ vs.* CD56^−^ IL-15 DCs: *P* = 0.03). Furthermore, intracellular cytokine staining revealed that both CD56*^+^* and CD56^−^ IL-15 DCs expressed considerable levels of granzyme B, albeit that the CD56*^+^* fraction expressed significantly more granzyme B than the CD56^−^ DC subset (Figure 5B; ΔMFI for intracellular granzyme B in CD56*^+^ vs.* CD56^−^ IL-15 DCs: 33.0±10.7 *vs.* 23.3±7.8; *P* = 0.004). In line with this, we observed a superior granzyme B secretory capacity of CD56^+^ IL-15 DCs (1121.0±353.3 pg/mL) over their CD56^−^ counterparts (452.3±117.0 pg/mL) (Figure 5C; *P* = 0.047). Strikingly, whereas intracellular levels of TRAIL and granzyme B were increased, perforin expression was found to be absent (Figure 5B).

### The cytotoxic activity of IL-15 DCs predominantly relies on granzyme B

**Figure 6:**
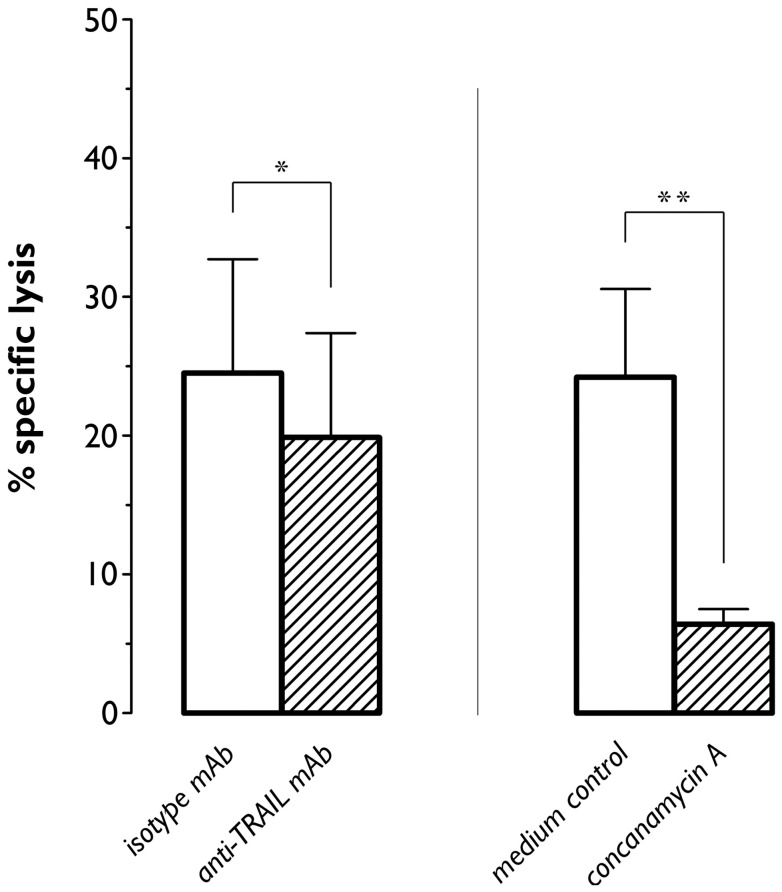
Inhibition of CD56^+^ IL-15 DC-mediated cytotoxicity by neutralizing anti-TRAIL mAbs and concanamycin A. Matured CD56^+^ IL-15 DCs were co-cultured with PKH67-labeled K562 target cells at an E:T ratio of 50:1 in the presence of either anti-TRAIL blocking mAb (left) or the granule exocytosis inhibitor concanamycin A (right). Parallel experiments were performed using TRAIL isotype-matched control mAb and medium control devoid of concanamycin A, respectively. Lysis of target cells was determined after overnight incubation using a flow cytometry-based cytotoxicity assay, as described above. Results are expressed as mean (± SEM) percentages of specific target cell lysis. Data are from 5 (for TRAIL) and 10 (for concanamycin) independent experiments. *, *P*<0.05; **, *P*<0.01.

Based on the data above, we envisaged two putative mechanisms underlying the cytotoxic action of CD56^+^ IL-15 DCs: TRAIL- and/or granzyme B-dependent apoptosis. To further dissect the relative contributions of these pathways to the pro-apoptotic effect of CD56^+^ IL-15 DCs on K562 cells, we performed cytotoxicity blocking experiments using anti-TRAIL neutralizing antibodies and concanamycin A, a selective inhibitor of vacuolar-type H^+^-ATPase that prevents acidification and degranulation of perforin/granzyme-containing cytotoxic granules. Since cytotoxicity of CD56^+^ IL-15 DCs against K562 cells was most prominent at high E:T ratios, we chose the E:T ratio of 50:1 for all further blocking experiments. As shown in Figure 6, neutralization of TRAIL activity resulted in a net decrease in cytotoxicity by 4.6±0.7% as compared to isotype control, corresponding to a 22.5±2.8% inhibition (Figure 6; *P* = 0.03). Upon incubation with concanamycin A, cytotoxicity of CD56^+^ IL-15 DCs against K562 was reduced from 24.2±6.3% (control medium) to 6.4±1.1%, corresponding to a 62.9±6.1% inhibition (Figure 6, *P* = 0.001). Taken together, these results confirmed that granzyme B and, to a small extent, TRAIL participated in the observed cytotoxicity of IL-15 DCs.

## Discussion

Dendritic cells, the quintessential antigen-presenting cells of the human immune system, have attracted much interest for active, specific immunotherapy of cancer over the years [32]. Despite some clinical successes, there is a general consensus that DC-based anti-tumor immunotherapy has not yet fulfilled its full therapeutic potential and that there remains considerable room for improvement, especially when it comes to optimizing the immunostimulatory activity of the DCs used for clinical application [32], [33]. Due to their potent immunostimulatory properties, monocyte-derived DCs generated in the presence of GM-CSF and IL-15 (IL-15 DCs) have been advocated as promising new vehicles for DC-based immunotherapy [17], [21]–[23]. In this study, we reveal for the first time that IL-15 DCs, in addition to a robust capacity for tumor antigen presentation, possess tumor cell killing potential. Our findings thus establish a previously unrecognized ‘killer DC’ function for IL-15 DCs, providing further support to their application in DC-based cancer immunotherapy protocols.

Although a subset of IL-15 DCs expresses the archetypal NK cell marker CD56 [34], we found no evidence for a further phenotypic overlap between IL-15 DCs and NK cells, nor could these cells be identified as the human homologue of murine NKDCs [4]–[9]. Our phenotypic data unequivocally establish that IL-15 DCs are genuine monocyte-derived DCs despite the rather unconventional expression of CD56. Perhaps the most compelling evidence for this comes from our cell sorting experiment in which CD14^+^ monocytes were flow sorted to ultra-high purity and then subjected to IL-15 DC differentiation. In this experiment, we showed that CD56^+^ IL-15 DCs can also be differentiated from a virtually pure, FACS-sorted CD14^++^CD16^−^ monocyte starting population, thus confirming that these cells are truly monocyte-derived and not related to NK cells. In addition, CD56^+^ IL-15 DCs fall within the flow cytometric scatter gate of DCs, but not of lymphocytes. The co-expression of the myeloid DC lineage markers BDCA-1 and CD11c along with the absence of CD7 expression, which allows their accurate discrimination from NK cells [26], [35], lends further support to the notion that IL-15 DCs are unrelated to NK cells in spite of their partial positivity for CD56.

To corroborate these phenotypic data and to confirm that IL-15 DCs also functionally qualify as DCs, we performed an allo-MLR as well as an antigen presentation assay. Both CD56^+^ and CD56^–^ IL-15 DCs are able to stimulate allogeneic T cell proliferation, thereby fulfilling one of the basic functional criteria for being qualified as DCs [29]. Furthermore, in this study, we also show that IL-15 DCs, as would be expected from DCs, are capable of processing and presenting the WT1 tumor antigen [30] to CD8^+^ T cells. Together with previous observations from our group and others [17], [21], [22], these data confirm that IL-15 DCs are “authentic” myeloid DCs not only from the phenotypic but also from the functional point of view. Strikingly, in the WT1 antigen presentation assay, CD56^+^ IL-15 DCs were found to have a superior antigen-presenting capacity over their CD56^–^ counterparts. Both fractions had comparable expression of the WT1 protein following electroporation (data not shown), suggesting that CD56^+^ DCs have a higher intrinsic ability to process and present endogenously synthesized antigen to T cells. Although the precise functional role of CD56 on DCs remains to be elucidated, the above data suggest that the expression of CD56 on DCs is linked with superior immunostimulatory activity. This mirrors the situation in NK cells and CD56-expressing T cells where CD56 expression and antigen density correlate with activation status and enhanced immune function [1], [36]–[43]. Further support for this statement comes from the phenotypic data presented in Table 1, which show that CD56^+^ IL-15 DCs are in a more differentiated and activated modus as compared to their CD56^–^ counterparts.

The observation that CD56^+^ IL-15 DCs, in addition to being potent allostimulatory and antigen-presenting cells, are endowed with a cytotoxic capacity is a novel finding that adds to the growing body of evidence that DCs can adopt an “unconventional” cytotoxic effector function and act as killer cells (reviewed in [10]–[12], [14]). Among the stimuli capable of triggering such ‘killer DC’ function are type I and II IFNs [44]–[49], TLR ligands [13], [48], [50]–[53] and, as shown here and in another recent study, IL-15 [54]. The fact that IL-15, a known growth factor for NK cells [34], was used in this study for DC differentiation as well as the fact that IL-15 DCs were found to be cytotoxic against the NK prototype target K562 [25], prompted us to perform rigorous culture purity assessments in order to exclude the possibility that the observed cytotoxic effects were due to the presence of contaminating NK cells. Based on the model proposed by Stary *et al.* [53], at least 10% of NK cell contaminants would have been needed to account for the cytotoxic activity reported in the present study. The trace contamination (<1%) of IL-15 DC cultures by lymphocytes was thus far too low to account for the observed cytotoxic effects and was therefore considered negligible [53]. This was also further supported by the finding that neither CD56^−^ nor CD56^+^ IL-15 DC preparations had cytotoxic activity against the U937 cell line, another well-recognized NK-sensitive target [25], [31].

The lack of cytotoxicity against U937 identifies a second point of difference between IL-15 DCs and NK cells, in addition to the finding that these cells do not share any phenotypic resemblance except for CD56 surface expression. Indeed, as discussed above, IL-15 DCs do not bear any other NK cell-associated surface markers, such as NKG2D or NCRs. The mechanism underlying the ability of NK cells to induce U937 cell death has been recently identified as being NCR-mediated [31], likely explaining the absent cytotoxic activity of IL-15 DCs against U937 cells. Another striking dissimilarity between IL-15 DCs and NK cells that merits further discussion is their differential pattern of cytotoxicity against the K562 cell line. While NK cells are strong and rapid inducers of K562 cell death, the anti-K562 cytotoxic activity of IL-15 DCs occurs only in the higher E:T range and with much slower dynamics. Interestingly, this intrinsically lower lytic potential has also been reported in other ‘killer DC’ studies and thus appears to be a common feature that distinguishes killer DCs from “classical” cytotoxic effector cells such as NK cells [46], [52], [53], [55]–[57]. The observation that IL-15 DCs display a distinct lytic profile further supports our view that these cells, despite the non-conforming expression of CD56, should be regarded as *bona fide* DCs endowed will killing potential and not as NK cells with antigen-presenting function [2].

An important finding from this study is that IL-15 killer DCs do not induce cell death of tumor antigen-specific T cells, suggesting that their cytotoxic action is tumor-selective. This is especially noteworthy in view of recent data from Luckey *et al.*, who showed that murine killer DCs are capable of eliminating allergen-specific T cells through a TNF-α-dependent mechanism and, as such, of preventing mice from developing allergic contact dermatitis [58]. In line with this, murine CD8^+^ DCs have been previously shown to be capable of inducing T cell apoptosis through the Fas/FasL pathway [59]. DC-mediated killing of T cells has also been demonstrated in the context of HIV infection [11], [60]. Evidently, the possibility of T cell killing would represent a major obstacle to the exploitation of killer DCs for cancer immunotherapy. Our data, however, indicate that T cell-directed cytotoxicity is not a general feature of killer DCs. This is consistent with the emerging view that killer DCs are a heterogeneous population, containing subsets that are preferentially tumoricidal as well as others that appear to be more biased toward a tolerogenic profile (e.g. through their ability for T cell killing) [10].

This heterogeneity also applies to the different cytotoxic effector mechanisms that can be used by killer DCs. FasL and TNF-α, previously described as key components of the lytic armamentarium of killer DCs [10], [11], [45], [59], are not found to be expressed on the IL-15 DC surface, thus arguing against their possible involvement in IL-15 DC-mediated killing. Although they lack membrane expression of TRAIL, IL-15 DCs – in particular the CD56^+^ fraction – harbor an internal pool of TRAIL molecules. Nevertheless, TRAIL neutralization resulted only in a marginal reduction of the lytic activity of CD56^+^ IL-15 DCs against K562 cells, indicating that TRAIL is not a major contributor to the cytotoxic action of these DCs. This is in contrast to several other studies that implied an important role for this death receptor ligand in DC-mediated cytotoxicity [44], [46], [48], [53]. Our results point to granzyme B-induced apoptosis as the main cell death pathway used by IL-15 DCs. The presence of intracellular granzyme B deposits in IL-15 DCs was ascertained by direct gating on the DC population on the basis of a combination of scatter profile and CD11c positivity. The functional importance of this expression was further supported by the capacity of IL-15 DCs to release granzyme B extracellularly and ultimately confirmed by the profound reduction of their cytotoxic activity using concanamycin A, which is commonly used to inhibit the perforin/granzyme B cytotoxic pathway [13], [53]. Intriguingly, we were unable to reveal expression of perforin in IL-15 DCs. This observation is in contrast to the study of Stary *et al.* in which TLR7/8-stimulated blood myeloid DCs were found to express both perforin and granzyme B [53], but is congruent with a recent report showing that mouse plasmacytoid DCs can kill in a granzyme B-dependent, perforin-independent fashion [13]. Although puzzling at first sight, the discordant expression of perforin and granzyme B apparently does not preclude IL-15 DCs from inducing K562 cell death. This complements the notion that granzyme B-induced apoptosis can still occur in the absence of perforin, although not with the same efficiency or rapidity [61]–[63]. The lack of perforin expression in IL-15 DCs may thus provide a plausible explanation for their differential lytic profile as compared to “classical” cytotoxic effector cells such as NK cells, which typically contain high levels of both perforin and granzyme B enabling them to induce rapid target cell death [63], [64].

In conclusion, we show here that IL-15 can drive the functional repertoire of human monocyte-derived DCs toward a killer DC profile. This study showcases the considerable potential for phenotypic and functional flexibility of human DCs and provides new converging evidence of the possibility that DCs can adopt a cytotoxic effector function. The observation that IL-15 DCs, in addition to being potent tumor antigen-presenting cells, are endowed with tumoricidal potential provides further strong support to the implementation of IL-15 DCs in DC-based anti-tumor immunotherapy strategies and to the use of IL-15 as an immunostimulatory adjunct in cancer therapy.

## Supporting Information

Figure S1 **Kinetics of CD56 expression on IL-15 DCs.** FACS-purified (>99.9% purity) CD14^+^ monocytes were cultured for 7 days with GM-CSF and IL-15 and analyzed by flow cytometry at the time points indicated for co-expression of CD11c/CD56 (right panels). Samples were stained in parallel with CD11c and an isotype-matched control mAb for CD56 to allow proper gate setting (left panels). Data shown are from a single donor and are representative of two separate experiments.
(TIF)

Figure S2 **Lack of cytotoxicity by CD56^+^ and CD56^−^ IL-15 DCs against the U937 target cell line.** Residual viability of PKH67-labeled U937 cells after overnight incubation in the absence or presence of either CD56^−^ (□) or CD56^+^ (▪) mature IL-15 DCs at an E:T ratio of 50:1. Viability was determined by flow cytometric quantitation of the percentage of PI^−^/Annexin-V^−^ – cells within the PKH67^+^CD11c^−^ gate. Bars represent mean (± SEM) percentages of viable cells from three independent experiments.
(TIF)
