# Correction: Interleukin-15-Induced CD56^+^ Myeloid Dendritic Cells Combine Potent Tumor Antigen Presentation with Direct Tumoricidal Potential

**DOI:** 10.1371/annotation/4f22e7e4-4882-43fa-abf1-1c888adbed15

**Published:** 2014-01-22

**Authors:** Sébastien Anguille, Eva Lion, Jurjen Tel, I. Jolanda M de Vries, Karen Couderé, Phillip D. Fromm, Viggo F. Van Tendeloo, Evelien L. Smits, Zwi N. Berneman

There were multiple errors and omissions in the Funding Statement. The Funding Statement should read: 

"This work was supported in part by research grants of the Research Foundation Flanders (FWO Vlaanderen, www.fwo.be), the Belgian Foundation against Cancer (Stichting tegen Kanker, www.kanker.be), the Methusalem program of the Flemish Government attributed to Prof Herman Goossens (University of Antwerp, Belgium), the Interuniversity Attraction Pole program (IAP #P6/41) of the Belgian Government and the Belgian Hercules Foundation (www.herculesstichting.be). SA is a former PhD fellow of the Research Foundation Flanders and currently holds an Emmanuel van der Schueren Fellowship of the Flemish League against Cancer (Vlaamse Liga tegen Kanker, www.tegenkanker.be). SA was awarded a 2012 Endeavour Research Fellowship by the Department of Industry, Innovation, Science, Research and Tertiary Education of the Australian Government (www.innovation.gov.au). SA also received financial support from the Belgian Foundation against Cancer and the Belgian public utility foundation VOCATIO (www.vocatio.be). EL was supported by a PhD grant of the Institute for the Promotion of Innovation through Science and Technology (IWT, www.iwt.be) and by an Emmanuel van der Schueren Fellowship of the Flemish League against Cancer. JT was supported by a grant from The Netherlands Organization for Scientific Research (NWO ZonMW, www.zonmw.nl). ELS is a post-doctoral fellow of the Research Foundation Flanders. The funders had no role in study design, data collection and analysis, decision to publish or preparation of the manuscript."

