# Supplementary figures and images for: Interleukin-15-Induced CD56+ Myeloid Dendritic Cells Combine Potent Tumor Antigen Presentation with Direct Tumoricidal Potential
Source: PLoS One. 2012 Dec 28;7(12):e51851. doi: 10.1371/journal.pone.0051851 (PMC3532168; doi:10.1371/journal.pone.0051851)

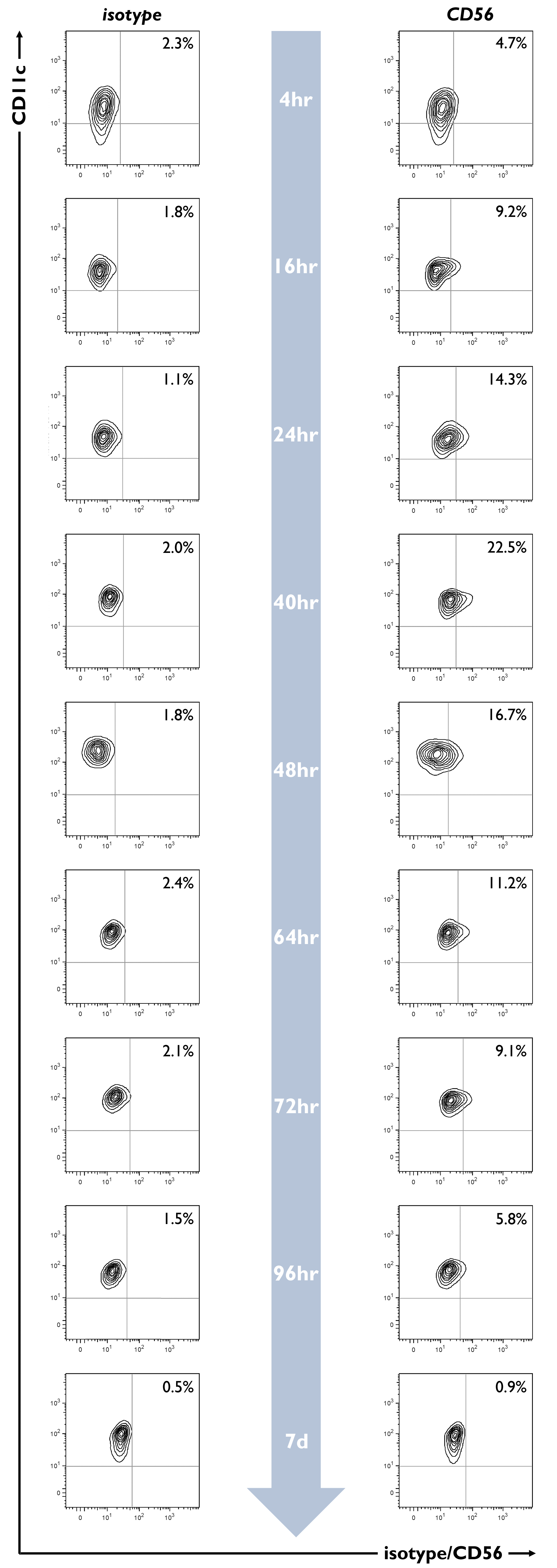

Supplement: Figure S1 — Kinetics of CD56 expression on IL-15 DCs. FACS-purified (>99.9% purity) CD14+ monocytes were cultured for 7 days with GM-CSF and IL-15 and analyzed by flow cytometry at the time points indicated for co-expression of CD11c/CD56 (right panels). Samples were stained in parallel with CD11c and an isotype-matched control mAb for CD56 to allow proper gate setting (left panels). Data shown are from a single donor and are representative of two separate experiments. (TIF) [file pone.0051851.s001.tif]

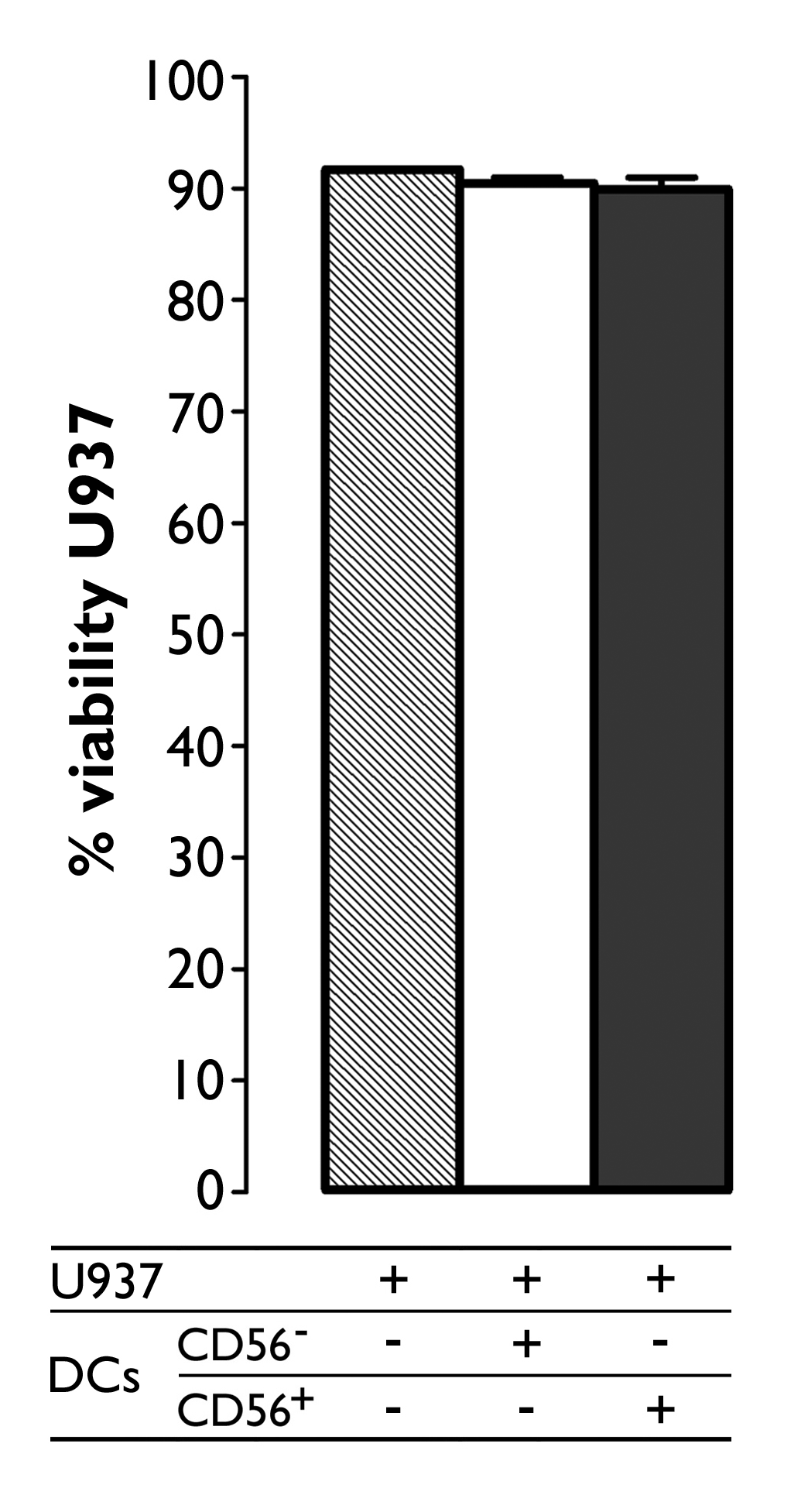

Supplement: Figure S2 — Lack of cytotoxicity by CD56+ and CD56− IL-15 DCs against the U937 target cell line. Residual viability of PKH67-labeled U937 cells after overnight incubation in the absence or presence of either CD56− (□) or CD56+ (▪) mature IL-15 DCs at an E:T ratio of 50:1. Viability was determined by flow cytometric quantitation of the percentage of PI−/Annexin-V− – cells within the PKH67+CD11c− gate. Bars represent mean (± SEM) percentages of viable cells from three independent experiments. (TIF) [file pone.0051851.s002.tif]
